# Calibration and Cross-Validation of the ActiGraph wGT3X+ Accelerometer for the Estimation of Physical Activity Intensity in Children with Intellectual Disabilities

**DOI:** 10.1371/journal.pone.0164928

**Published:** 2016-10-19

**Authors:** Arlene M. McGarty, Victoria Penpraze, Craig A. Melville

**Affiliations:** 1 Institute of Health & Wellbeing, College of Medical, Veterinary and Life Sciences, University of Glasgow, Glasgow, Scotland; 2 School of Life Sciences, College of Medical, Veterinary and Life Sciences, University of Glasgow, Glasgow, Scotland; Universidad Europea de Madrid, SPAIN

## Abstract

**Background:**

Valid objective measurement is integral to increasing our understanding of physical activity and sedentary behaviours. However, no population-specific cut points have been calibrated for children with intellectual disabilities. Therefore, this study aimed to calibrate and cross-validate the first population-specific accelerometer intensity cut points for children with intellectual disabilities.

**Methods:**

Fifty children with intellectual disabilities were randomly assigned to the calibration (*n* = 36; boys = 28, 9.53±1.08yrs) or cross-validation (*n* = 14; boys = 9, 9.57±1.16yrs) group. Participants completed a semi-structured school-based activity session, which included various activities ranging from sedentary to vigorous intensity. Direct observation (SOFIT tool) was used to calibrate the ActiGraph wGT3X+, which participants wore on the right hip. Receiver Operating Characteristic curve analyses determined the optimal cut points for sedentary, moderate, and vigorous intensity activity for the vertical axis and vector magnitude. Classification agreement was investigated using sensitivity, specificity, total agreement, and Cohen’s kappa scores against the criterion measure of SOFIT.

**Results:**

The optimal (AUC = .87−.94) vertical axis cut points (cpm) were ≤507 (sedentary), 1008−2300 (moderate), and ≥2301 (vigorous), which demonstrated high sensitivity (81−88%) and specificity (81−85%). The optimal (AUC = .86−.92) vector magnitude cut points (cpm) of ≤1863 (sedentary), 2610−4214 (moderate), and ≥4215 (vigorous) demonstrated comparable, albeit marginally lower, accuracy than the vertical axis cut points (sensitivity = 80−86%; specificity = 77−82%). Classification agreement ranged from moderate to almost perfect (κ = .51−.85) with high sensitivity and specificity, and confirmed the trend that accuracy increased with intensity, and vertical axis cut points provide higher classification agreement than vector magnitude cut points.

**Conclusions:**

This study provides the first valid methods of interpreting accelerometer output in children with intellectual disabilities. The calibrated physical activity cut points are notably higher than existing cut points, thus raising questions on the validity of previous low physical activity estimates in children with intellectual disabilities that were based on typically developing cut points.

## Introduction

Physical activity is associated with many physical and mental health benefits in children, such as reduced body mass index (BMI), increased bone health, reduced risk of metabolic syndromes, and lower rates of depression [1–3]. Furthermore, childhood physical activity is a determinant of physical activity in adulthood, therefore it is important that positive behaviours are promoted in children [4, 5]. This is of primary importance in children with intellectual disabilities as this population participate in low levels of activity and have a higher prevalence of negative health outcomes in comparison with their typically developing peers [6, 7]. Therefore, there is a need to increase our understanding of physical activity in this population and develop effective interventions to increase activity levels. In accordance with best practice guidelines on the development of effective interventions, research has to be based on valid measurement of various parameters of activity, such as intensity, frequency, and duration [8, 9]. This will increase our understanding of dose-response relationships and determinants of activity, from which interventions can be developed.

Free-living physical activity can be measured using subjective methods (such as questionnaires or activity logs) or objective methods (such as accelerometers and pedometers). Subjective measures provide feasible methods for collecting data on various parameters of physical activity, but this high feasibility is at a cost to validity. Due to the cognitive demands associated with subjective measures, this lower validity is particularly apparent in children, which will be exacerbated in children with intellectual disabilities [10 11]. Of the commonly used objective measures, pedometers are affected by abnormal gait patterns and heart monitors affected by atypical heart rates, both of which are prevalent in children with intellectual disabilities [11–14]. Accelerometers therefore provide the most comprehensive and feasible method of measuring parameters of activity in children with intellectual disabilities.

Accelerometers are small, non-intrusive devices that measure acceleration of the body during movement. In general, older accelerometers measure acceleration on the vertical axis of the body, but advantaging technology now enables acceleration to be measured on up to three planes [15]. Acceleration signals are post-processed and converted into arbitrary activity “counts” for the vertical axis or combined three axes (vector magnitude), which can be calibrated to provide biologically meaningful data, such as physical activity intensity [16].

Accelerometer calibration is age- and population-specific due to the effects of maturation and between group differences, such as cardiorespiratory fitness [17, 18]. Therefore, generalising intensity cut points between populations introduces systematic measurement error and reduces validity. However, accelerometers have not been calibrated for children with intellectual disabilities, thus raising questions on the validity of generalising typically developing cut points to children with intellectual disabilities due to movement and metabolic differences between these groups [18–20]. As a result, the calibration of accelerometer cut points is an important next step is the development of a valid knowledge-base on the physical activity behaviours of children with intellectual disabilities, from which effective interventions can be developed.

Therefore, the aims of this study are to: 1) calibrate ActiGraph wGT3X+ vertical axis and vector magnitude cut points for the estimation of sedentary, moderate, and vigorous intensity activity in children with intellectual disabilities, and; 2) cross-validate the developed cut points in a sub-sample of children with intellectual disabilities.

## Methods

### Ethical Consideration

This study was approved by the Medical, Veterinary, and Life Sciences College Ethics Committee, University of Glasgow. Prior to participation, written informed consent was obtained from participants and parents.

### Participants

Five additional support needs primary schools in the West of Scotland, which were specifically for children with mild to moderate intellectual disabilities, were used for recruitment and data collection. To be eligible for participation, children had to be aged 8 to 11 years, have intellectual disabilities, and be independently ambulatory. No data was collected on the aetiology of disabilities of participants due to feasibility reasons, e.g. the need to conduct IQ tests. In total, 86 information packs were handed out to eligible children (60 boys, 26 girls), which resulted in 50 children taking part, representing a recruitment rate of 58.14%.

### Protocol

A semi-structured physical activity session was designed specifically for this calibration study. A semi-structured protocol was used to increase and ecological validity and ensure that children participated in sufficient activity at each intensity. The session content was developed based on previous field-based calibration and validation research conducted in typically developing children and discussions with teachers to ensure the use of appropriate and familiar activities. In addition, the energy expenditure compendium for youth was used as a guide to inform the inclusion of activities that were of the required activity intensities [21]. The semi-structured session was developed with four main phases: warm-up, instruction games, obstacle games, and team games (Table 1). Although all sessions included these four phases, the semi-structured nature of the protocol enabled the activities to be adapted to suit the participants’ level of ability.

**Table 1:** Description of activity protocol.

Phase	Description of main activities	Approximate time (min)	Intensity
Warm up	Active stretches, walking, jogging	10	Light/moderate
Instruction games	Moving around the hall following instructions from the researcher, e.g. star jump, touch floor.	10	Moderate
Obstacle games	Short courses included various aerobic activities, e.g. agility ladder, zig-zag cones, bench jumps.	10	Vigorous
Team games	Dodge ball and tag.	15	Vigorous

### Measures

#### Anthropometric

Height was measured to the nearest 0.1 centimetre using a stadiometer (Seca Scales, Hamburg, Germany) and weight measured to the nearest 0.1 kilogram using digital scales (Seca Scales, Hamburg, Germany). Measurements were conducted twice to produce a mean value whilst participants were wearing light clothing and no shoes.

#### Accelerometry

Physical activity was measured using the ActiGraph wGT3X+ accelerometer (ActiGraph, LLC, Pensacola, FL, USA). This small, lightweight device (46 × 33 × 15 mm, 19 g) measures acceleration during movement across the vertical, horizontal, and perpendicular axes. Prior to the session, the accelerometers were initialized to record accelerations at a sampling frequency of 30Hz. Participants wore one device on their right hip at the iliac crest, attached using an elastic belt, for the duration of the activity session.

#### Criterion measure: Direct observation

The System for Observing Fitness Instruction Time (SOFIT) is a momentary time sampling direct observation tool [22]. The ‘student activity’ element of this tool categorises physical activity behaviours as: lying down, sitting, standing, walking, and very active, and has been validated for typically developing children and children with intellectual disabilities [23–25]. Activity was coded every 20 seconds using 10-second observe/record intervals, yielding 3 observations per minute, and was paced using pre-recorded audio MP4 files [26]. If the participant was transitioning from one activity to another at the end of the observe interval, the activity was recorded as the higher code; for example, if transitioning from lying down to sitting, the activity was coded as sitting [26].

To ensure the SOFIT data were reliable and valid, three raters (AMMcG, VP, CAM) were involved in data coding. Raters undertook eight hours of classroom-based training which included understanding coding procedures and definitions, video analysis practice, and validity assessment [26]. A combined accuracy of 86% was achieved with the gold standard assessment video, which exceeds the minimum recommendation of 80% [26]. Data from one rater (AMMcG) was used for data collection, with the other raters (VP & CAM) used to assess field-based reliability. Initial reliability was established using data from two randomly selected participants from session one, with inter- and intra-rater reliability scores of 79% and 89%, respectively, achieved. At the midpoint of data collection, reliability was further investigated using two randomly selected participants from session three, with inter- and intra-rater reliability of 85% and 91% achieved, respectively, thus confirming the data collected by the lead rater was reliable.

#### Management of data

Accelerometer data for the vertical axis and vector magnitude were downloaded using ActiLife version 6.11.5 software (ActiGraph LLC, Pensacola) in 10-second epochs. Video data was time matched to the accelerometer data to ensure the SOFIT coding started at the beginning of a 10-second accelerometer epoch. Two consecutive 10-second vertical axis and vector magnitude epochs were summed using an Excel macro to correspond with one 20-second SOFIT epoch. This produced data in the following formats: vertical axis counts (counts/20-sec), vector magnitude counts (counts/20-sec), and SOFIT classification (score/20-sec). Data were then screened for spurious scores and epochs where the participant left the gym hall were excluded from the analysis. Data in this format were used for all analyses. Prior to conducting the calibration analyses, data for 14 participants were removed to enable cross-validation analyses, with two participants randomly selected from each of the seven sessions, resulting in data from 36 participants being used for calibration.

#### Statistical analysis

All statistical analyses were conducted using SPSS 22 IBM statistical package (SPSS IBM, New York, NY, USA). Descriptive statistics (mean ± SD) were calculated for all participant and session variables. Independent samples t-tests were additionally conducted to test for differences in age, height, weight, and BMI between the calibration and cross-validation groups. Receiver operating characteristic (ROC) curve analyses were conducted to determine the optimal cut points for the classification of sedentary, moderate, and vigorous intensity activity. ROC curve analysis quantifies the relationship between positive and negative scores for continuous data and allows a cut point to be identified which best discriminates between two conditions [27]. A “positive” score represents the condition of interest, whereas a “negative” score is not the condition of interest.

ROC curves were interpreted using sensitivity, specificity, and the area under the curve (AUC) of the ROC curve. Sensitivity is the accuracy of a cut point to correctly classify activity intensity (true positive) whereas specificity is the accuracy of a cut point to exclude data which is not of the specified intensity (false positive). In addition, the AUC gives a statistical representation of the accuracy of the optimal cut point. The AUC is the average true positive classification rate, independent of false positive classifications. Therefore, a cut point which perfectly classifies all scores will have an AUC of 1.0, with a cut point equivalent to chance having an AUC of .50. The AUC scores will be interpreted using the following scale: ≥ .90 is excellent, .80-.89 is good, .70-.79 is fair, and < .70 is poor [28]. In line with previous accelerometer calibration studies, the aim of this ROC curve analysis was to identify the cut point which maximises both sensitivity and specificity. This optimal cut points was identified by manually applying Youden’s index to all identified cut points: Youden’s index = maximum (sensitivity + sensitivity– 1).

For the ROC curve analysis, SOFIT scores were converted into binary scores, with binary code 1 representing a positive score and binary code 0 representing a negative score. Separate analyses were conducted for the calibration of the sedentary, moderate, and vigorous cut points for the vertical axis and vector magnitude, with the vigorous cut point providing the upper boundary for the moderate intensity cut point. Table 2. summarises the binary codes used for each analysis.

**Table 2:** Summary of the conversion of SOFIT categories into binary codes for ROC curve analysis.

Activity intensity	SOFIT categories	
	Binary code 1 (positive)	Binary code 0 (negative)
Sedentary	1, 2	3, 4, 5
Moderate	4, 5	1, 2, 3
Vigorous	5	1, 2, 3, 4

SOFIT categories: lying down = 1; sitting = 2; lying down = 3; walking = 4; very active = 5

For cross-validation, classification agreement between SOFIT and the calibrated cut points was investigated using sensitivity, specificity, total agreement percentages, and Cohen’s kappa scores. Kappa scores (κ) provide a statistical measure of agreement, accounting for agreements which may occur by chance, and will be interpreted using the following scale: < .00 is less than change agreement, .00−.20 is slight agreement, .21−.40 is fair agreement, .41−.60 is moderate agreement, .61−.80 is substantial agreement, and .81−1.00 is almost perfect agreement [29].

## Results

Descriptive statistics for all participants are presented in Table 3. There were no significant differences in age (t = -.13, df = 48, p > .01, 95% CI -.74, .65) height (t = .08, df = 48, p > .01, 95% CI -.05, .06), weight (t = -.06, df = 48, p > .01, 95% CI -6.78, 6.38), or BMI (t = -.32, df = 48, p > .05, 95% CI -2.81, 2.05) between the participants in the calibration and cross-validation groups, suggesting the cross-validation sample is representative of the calibration group. Seven activity sessions were conducted, with descriptive data for each session presented in Table 4. In total, 1057 minutes of data were used for the calibration analyses and 382 minutes used for cross-validation. This equates to 256, 257, 338, and 206 minutes for sedentary, moderate, light, and vigorous intensity activity for calibration and 92, 82, 107, and 101 minutes for each intensity, respectively, for cross-validation.

**Table 3:** Descriptive statistics (mean ± SD) of all participants and participants in the calibration and cross-validation groups.

Characteristic	Participants		
	All participants (n = 50)	Calibration (n = 36)	Cross-validation (n = 14)
Age (yrs)	9.54 ± 1.09	9.53 ± 1.08	9.57 ± 1.16
Height (m)	1.43 ± .09	1.43 ± .09	1.43 ± .08
Weight (kg)	39.33 ± 10.28	39.28 ± 11.46	39.48 ± 6.69
BMI (kg/m^2^)	19.09 ± 3.80	18.99 ± 4.15	19.37 ± 2.81
Sex	M = 37, F = 13	M = 28, F = 8	M = 9, F = 6

**Table 4:** Descriptive statistics on session duration, participants, and percentage of the session spent in each SOFIT category.

Session	School	Session duration (min)	Lying down (%)	Sitting (%)	Standing (%)	Walking (%)	Very active (%)	Boys (n)	Girls (n)
1	1	25	3.96	11.08	27.70	36.15	21.12	7	1
2	2	34	6.25	26.72	17.03	29.66	20.34	5	3
3	2	38	4.50	28.23	27.93	22.02	17.32	4	5
4	3	40	4.06	12.19	26.40	24.37	32.97	7	0
5	3	29	1.64	21.51	21.67	41.87	13.30	7	0
6	4	27	3.27	22.67	17.38	29.97	26.70	3	2
7	5	16	3.47	29.86	11.11	31.94	23.61	5	1

Note: two sessions were conducted at schools 2 and 3 for feasibility reasons due to the higher number of participants recruited

The sedentary, moderate, and vigorous count boundaries developed (cpm) were ≤ 507, 1008−2300, and ≥ 2301 for the vertical axis and ≤ 1863, 2610−4214, and ≥ 4215 for vector magnitude, respectively. As shown in Table 5, these cut points exhibit high sensitivity (80−88%) and specificity (77−85%) scores, with the accuracy of the cut points increasing with intensity (AUC = .86−.94). For the vertical axis and vector magnitude, the sedentary cut points demonstrated good classification accuracy, with the moderate and vigorous cut points demonstrating excellent AUC scores. The high sensitivity and specificity scores illustrate that these cut points should limit misclassifications, with the vertical axis cut points providing marginally higher accuracy than that vector magnitude cut points. ROC curve graphs for each cut point are presented in S1 Fig.

**Table 5:** Calibration and cross-validation statistics for the derived cut points.

	Cut point (counts/20-sec)	Cut point (cpm)	Calibration			Cross-validation			
			Sensitivity (%)	Specificity (%)	AUC (95% CI)	Sensitivity (%)	Specificity (%)	Total agreement (%)	κ (SE)
**Vertical axis**									
Sedentary	≤ 169	≤ 507	81	81	.87 (.86−.88)	93	83	85	.66* (.02)
Moderate	336−766	1008−2300	86	83	.92 (.91−.93)	75	96	90	.74* (.02)
Vigorous	≥ 767	≥ 2301	88	85	.94 (.93−.95)	93	95	94	.85* (.02)
MVPA	≥ 336	≥ 1008				91	95	93	.85* (.02)
**Vector magnitude**									
Sedentary	≤ 621	≤ 1863	80	77	.86 (.84−.87)	83	86	85	.63* (.03)
Moderate	870−1404	2610−4214	86	82	.92 (.91−.93)	60	89	79	.51* (.03)
Vigorous	≥ 1405	≥ 4215	85	82	.92 (.91−.93)	89	89	90	.74* (.02)
MVPA	≥ 870	≥ 2610				91	84	87	.75* (.02)

* Significant at p < .001

When cross-validated, the cut points demonstrated fair to almost perfect classification agreement (Table 5). The moderate intensity cut point for vector magnitude was the only cut point that demonstrated a substantially lower level of accuracy than expected, based on the calibration results. Consistent with the calibration findings, the vertical axis cut points demonstrated higher classification agreement, with classification agreement also increasing with activity intensity.

## Discussion

This study calibrated and cross-validate the first accelerometer cut points for the classification of sedentary, moderate, and vigorous intensity activity in children with intellectual disabilities, and empirically demonstrated the need for population-specific cut points. Due to the associated health outcomes of sedentary behaviour and MVPA, and the need for more standardised objective measurement [30, 31], the current recommendation is to use a vertical axis cut point of 100 cpm for sedentary and the Evenson et al. [32] cut point of ≥ 2296 cpm for MVPA in typically developing children [33, 34]. However, as the sedentary and MVPA cut points developed in this study are notably different to these recommended cut points, it is important that the measurement of physical activity in children with intellectual disabilities is viewed independent of typically developing research.

To further highlight the effect of generalising cut points, the lower boundary of many exiting moderate intensity cut points are higher than the upper boundary of the present moderate cut point, i.e. > 2300 cpm (Table 6). Similarly, the calibrated vigorous cut point of ≥ 2301 cpm is also lower than some existing moderate intensity cut points, i.e. the lower boundary for the moderate cut points developed by Puyau et al. [35], Treuth et al. [36], and Mattocks et al. [37], are > 2301 cpm. Therefore, if generalised to children with intellectual disabilities, potentially all moderate and vigorous intensity activity will be misclassified. Furthermore, this raises questions on the validity of existing literature which has quantified physical activity levels in children with intellectual disabilities by generalising cut points calibrated in typically developing children (e.g. Einarsson et al. [6] and Phillips & Holland [38]).

**Table 6:** Existing ActiGraph vertical axis and vector magnitude cut points calibrated for typically developing children.

Cut points	Sedentary	Moderate	Vigorous
**Vertical Axis**			
Puyau (2002)	0–799	3200–8199	≥ 8200
Treuth (2004)	0–100	3000–5200	≥ 5201
Freedson (2005)	0–500*	501–4000	4001–7600
Mattocks (2007)	0–100	3581–6129	≥ 6130
Evenson (2008)	0–100	2296–4011	≥ 4012
Pulsford (2011)	0–99	2241–3840	≥ 3841
Vanhelst (2011)	0–400	1901–3918	≥ 3919
Mackintosh (2012)	0–372	2161–4806	≥ 4807
Jimmy (2013)	n/a	1596–2315	≥ 2316
Romanzini (2014)	0–184	2428–3271	≥ 3272
Current study	0–507	1008–2300	≥ 2301
**Vector Magnitude**			
Santos-Lozano (2013)	0–2114*	2114–6547	6548–11490
Hanggi (2013)	0–180	> 3360 (MVPA)	
Jimmy (2013)	n/a	2952–3791	≥ 3792
Romanzini (2014)	0–720	3028–4447	≥ 4448
Current study	0–1863	2610–4214	≥ 4215

* Represents sedentary and light intensity

With the evolving technology of ActiGraph devices, the ability to measure three axes and calculate vector magnitude should theoretically increase the accuracy of capturing the dynamic activity behaviours of children; however, there is currently no consensus on this [30, 39, 40]. Consistent with the findings of the present study, previous empirical research has demonstrated that vector magnitude cut points do not provide a consistently higher level of validity than vertical axis cut points [41–43]. The moderate and vigorous vector magnitude cut points in the present study are similar to the existing cut points, which were all calibrated using the ActiGraph GT3X device (Table 6). Therefore, as third generation ActiGraph devices record higher count values than older devices (e.g. the GT1M), the device used for calibration could have contributed to the smaller variance identified with existing vector magnitude cut points [44, 45]. Furthermore, as most existing vertical axis cut points were calibrated using GT1M or AM7164 devices, the wGT3X+ device could have contributed to the lower vector magnitude cut points developed in the present study. With the large differences identified between the cut points calibrated in the presented study, it is important to further consider why such notable differences occurred, and additional factors which could have affected calibration.

As most of the activities used in previous calibration studies were structured and constant, the accelerometer will record more consistent counts and the criterion method will more accurately measure activity intensity [32, 41, 46–48]. The free-living design of the current protocol made it more difficult to discriminate between activity intensity, with the data recorded by SOFIT affected by transitions or epochs containing more than one intensity of activity. However, this protocol design will better account for the sporadic nature of children’s activity, thus increasing ecological validity.

As the criterion measure, the validity of the developed cut points is dependent on the accurate use of SOFIT. Although a criterion measure, there is some subjectivity in the classification of what is deemed “ordinary” walking, which will vary between children and may include activity which is not of a moderate intensity. This could have contributed to the lower moderate intensity boundaries, particularly in comparison with existing cut points not established using SOFIT. Furthermore, as SOFIT does not capture extraneous movements which may be detected by the accelerometer, such as foot tapping, this could at least partially account for the higher sedentary cut points calibrated [49]. There is also a lack of consensus on whether standing should be coded as sedentary activity [50].

As calibration is based on observed behaviours, these findings show that during the same biomechanical movements, children with intellectual disabilities produce a smaller acceleration, which could be a result of abnormal gait patterns [13, 14]. On the other hand, it is important to note that adults with intellectual disabilities have a significantly higher energy expenditure than adults without intellectual disabilities during the same laboratory-based activities [51]. Therefore, there is a need to further investigate biomechanical and physiological differences between children with and without intellectual disabilities, which could empirically identify population differences that could have contributed to the variance between the present cut points and existing cut points.

### Strengths and Limitations

This study was the first to calibrate and cross-validate accelerometer cut points for physical activity intensity in children with intellectual disabilities, which provides the first stages of establishing valid methods to interpret accelerometer output in this population. With vector magnitude calibration in its relative infancy, this study has placed measurement research in children with intellectual disabilities in line with the emerging research in typically developing children. The free-living design of this protocol enabled calibration and cross-validation to be conducted on representative activities, thus increasing ecological validity. Cross-validation was also in accordance with best practice recommendations, in that validation was conducted during a field-based protocol in a sample independent of the calibration group [52, 53]. Furthermore, although participation rates are generally low for health-related research involving children with intellectual disabilities, the high recruitment rate within this study is similar to that reported in previous research involving typically developing children.

Not without limitations, the semi-structured protocol resulted in between-session differences (Table 4); therefore, the data collected may not be fully representative of the study sample as a whole. More generally, a limitation with cross-validation is that it only estimates how valid the cut points will be in a sample similar to the calibration sample [54]. As cut points are generally age-specific, investigation into the effect of age on validity is important [34]. However, as the age range of the participants in the present study was relatively small, it was not possible to make inferences regarding the validity of these cut in children with intellectual disabilities who are younger or older than the included sample. Furthermore, as specific data were not collected regarding the aetiology of participant’s intellectual disabilities, it was not possible to investigate disability-related factors that could affect the generalisation of these cut points.

## Conclusions

This study was the first to calibrate and cross-validate population-specific accelerometer cut points for the estimation of physical activity intensity in children with intellectual disabilities, thus addressing a substantial gap in measurement research relating to this population. Overall, the cut points developed in this study show high sensitivity and specificity for the estimation of physical activity and sedentary behaviours in children with intellectual disabilities, with most demonstrating high classification agreement. With the trend in this study that lower physical activity cut points and higher sedentary cut points are required, in comparison with typically developing children, possible causes for these differences have been discussed. To further increase our knowledge on the validity of the developed cut points, additional field-based and longitudinal validation research needs to be conducted. Moving forward, it is important to consider whether future research may benefit from taking an additional step back to basics and increase our knowledge relating to the biomechanics of how children with intellectual disabilities move and further investigate physiological differences with typically developing children. This will help our understanding of population-specific factors which may have influenced calibration and will help inform the next phases of improving the validity of objectively measured physical activity in children with intellectual disabilities.

## Supporting Information

S1 Fig ROC curves and optimal cut points for sedentary, moderate, and vigorous intensity for vertical axis and vector magnitude counts. (DOCX)
