# Supplementary material for: Calibration and Cross-Validation of the ActiGraph wGT3X+ Accelerometer for the Estimation of Physical Activity Intensity in Children with Intellectual Disabilities
Source: PLoS One. 2016 Oct 19;11(10):e0164928. doi: 10.1371/journal.pone.0164928 (PMC5070820; doi:10.1371/journal.pone.0164928)
Supplement: S1 Fig — (DOCX) [file pone.0164928.s001.docx]

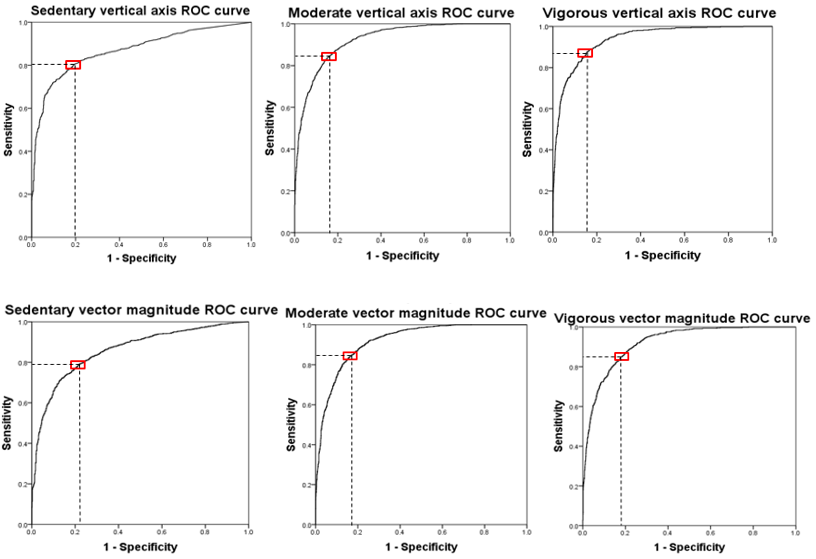


**Supporting information**. ROC curves and optimal cut points for sedentary, moderate, and vigorous intensity for vertical axis and vector magnitude counts
